# Dynamic changes in the pregnancy microbiome and their role in preterm birth

**DOI:** 10.3389/fcimb.2025.1683610

**Published:** 2025-12-19

**Authors:** Zhuojun Xie, Zhongsheng Chen, Guangyu Ma

**Affiliations:** 1 General Medicine Department, Clinical Medical College & Affiliated Hospital of Chengdu University, Chengdu University, Chengdu, China; 2 Department of General Surgery, The Fourth Affiliated Hospital of Harbin Medical University, Harbin, China; 3 Department of Obstetrics and Gynecology, The First Affiliated Hospital of Jinan University, Guangzhou, China

**Keywords:** gut microbiome, oral microbiome, preterm birth, probiotics, vaginal microbiome

## Abstract

Preterm birth (PTB) remains a leading cause of neonatal morbidity and mortality worldwide, posing significant challenges to maternal and child health. Recent advances have highlighted the critical role of the maternal microbiome-encompassing vaginal, gut, and oral microbial communities-n influencing pregnancy outcomes. This review comprehensively summarizes the dynamic changes of the pregnancy microbiome and elucidates its association with PTB. During healthy pregnancy, the vaginal microbiome is dominated by *Lactobacillus* with low diversity, while dysbiosis with fewer *Lactobacilli* and more anaerobes increases PTB risk. The gut microbiome also shifts, with reduced beneficial bacteria and more pro-inflammatory species linked to adverse outcomes. Changes in the oral microbiome and periodontal disease can promote systemic inflammation contributing to PTB. Microbial imbalance may trigger PTB through inflammation, immune changes, and microbial spread to the uterus. Targeting the microbiome via probiotics shows promise, but more clinical studies are needed. This review highlights the pregnancy microbiome as a key biomarker and intervention target to reduce PTB.

## 1 Introduction

Preterm birth (PTB) is a major global challenge in maternal and neonatal health, affecting around 15 million infants annually-approximately 11% of all births (Chawanpaiboon et al., 2019). It is a leading cause of mortality, accounting for 15% of child deaths and 35% of neonatal deaths (Saigal and Doyle, 2008). Common complications include bronchopulmonary dysplasia, respiratory distress syndrome, intraventricular hemorrhage, neurodevelopmental disorders, and necrotizing enterocolitis (Saigal and Doyle, 2008), which contribute significantly to under-five mortality. PTB can be classified into spontaneous PTB (40-45%), preterm premature rupture of membranes (PPROM) (25-30%), and medically or electively indicated preterm births (30-35%) (Goldenberg et al., 2008). The causes are multifactorial, involving intrauterine infection, immune dysregulation, genetic susceptibility, cervical insufficiency, uterine anomalies, and stress-related hormonal disruptions (Sykes et al., 2012; Silva et al., 2020; Mitrogiannis et al., 2023; Wei et al., 2024). These factors may act individually or synergistically to compromise pregnancy and precipitate premature labor.

The microbiome plays a critical role in health and disease, and its relevance during pregnancy has garnered increasing attention. During gestation, the maternal microbiome undergoes dynamic changes that can significantly influence both maternal and neonatal outcomes (Miao et al., 2025). The “pregnancy microbiome” includes microbial communities in the vagina, gastrointestinal tract, and oral cavity, along with their interactions with host physiology. Disruptions in microbial balance have been associated with an elevated risk of PTB (Bayar et al., 2020). For instance, reduced diversity or compositional changes in the vaginal microbiome, such as decreased *Lactobacillus* populations and increased levels of pathogenic bacteria, have been linked to a higher risk of PTB (Hyman et al., 2014; DiGiulio et al., 2015). Similarly, gut microbiome dysbiosis may contribute to PTB by influencing systemic inflammatory responses and immune regulation (Espinoza et al., 2002; López et al., 2016).

Understanding the complex relationship between the pregnancy microbiome and PTB is crucial for developing new preventive and therapeutic strategies. This review aims to comprehensively summarize the changes in the pregnancy microbiome and their impact on PTB, elucidating how dysbiosis influences PTB through mechanisms such as triggering inflammatory responses, modulating the immune system, and facilitating microbial migration. Special attention is given to the interactions between the microbiome and host immune and inflammatory pathways and how they affect the stability of the uterine environment. Additionally, strategies for maintaining or restoring a healthy microbiome to reduce PTB risk are explored, including dietary adjustments, the use of probiotics and prebiotics, and targeted interventions such as prophylactic antibiotics and microbiome transplantation.

## 2 The pregnancy microbiome: composition and dynamics

### 2.1 The vaginal microbiome

The female vaginal microecosystem is a complex network comprising the vaginal microbiota, host endocrine system, anatomical structures, and local immune defenses. Within this ecosystem, *Lactobacilli* play a crucial protective role as the dominant constituents of a healthy vaginal microbiota (Kwon and Lee, 2022). Among the *Lactobacillus* species found in the vaginal microbiota, *Lactobacillus iners*, *Lactobacillus crispatus*, *Lactobacillus gasseri*, and *Lactobacillus jensenii* are the most prevalent in healthy women, followed by *Lactobacillus acidophilus*, *Lactobacillus fermentum*, *Lactobacillus plantarum*, *Lactobacillus brevis*, *Lactobacillus casei*, and *Lactobacillus vaginalis*. Other species, such as *Lactobacillus delbrueckii*, *Lactobacillus salivarius*, *Lactobacillus reuteri*, and *Lactobacillus rhamnosus*, are isolated less frequently than the aforementioned species (Cribby et al., 2008). *Lactobacilli* maintain the vaginal acidic environment primarily by producing lactic acid, which inhibits pathogen growth (Doerflinger et al., 2014; Borges et al., 2014; Kovachev, 2018). Additionally, they prevent colonization by harmful microorganisms through competitive exclusion and produce antimicrobial compounds like hydrogen peroxide and bacteriocins, further enhancing vaginal defenses. Beyond these direct antimicrobial effects, *Lactobacilli* also interact with vaginal epithelial cells to modulate immune responses and strengthen local immunity.

The vaginal microecosystem of reproductive-aged women can be categorized into five main community state types (CSTs) based on the predominant bacterial genera and species (Ravel et al., 2011, 2011). Among these, CSTs I, II, III, and V are characterized by more than 90% dominance of *Lactobacilli*, collectively referred to as *Lactobacillus*-Dominated (LDOM) types. Specifically, CST I is predominantly occupied by *Lactobacillus crispatus*, CST II is primarily composed of *Lactobacillus gasseri*, CST III is dominated by *Lactobacillus iners*, and CST V mainly consists of *Lactobacillus jensenii*. In contrast, CST IV is characterized by a lower abundance of *Lactobacilli* and exhibits greater microbial diversity, primarily comprising strict anaerobes or facultative anaerobes, and is referred to as *Lactobacillus*-Depleted (LDEPL). CST IV is further subdivided into several subtypes, subtype IVA, primarily associated with bacteria linked to bacterial vaginosis (BV), subtype IVB, dominated by *Gardnerella* vaginalis, subtype IVC0, primarily consisting of *Prevotella*, subtype IVC1, mainly made up of *Streptococcus*, subtype IVC2, dominated by *Enterococcus*, subtype IVC3, primarily consisting of *Bifidobacterium*, and subtype IVC4, dominated by *Staphylococcus*. Although factors such as ethnicity and cultural background may influence the specific composition of the vaginal microbiota, the vaginal microecosystem of most healthy women is predominantly characterized by CSTs I, II, and V (Smith and Ravel, 2017).

The secretory phase of the menstrual cycle is characterized by high estrogen and progesterone levels, during which the vaginal microbiota tends to be relatively stable (Gajer et al., 2012). Pregnancy, with even higher and sustained elevations of these hormones, induces profound changes in the vaginal microenvironment that promote microbial stability. Elevated estrogen and progesterone stimulate glycogen synthesis and accumulation in vaginal epithelial cells, providing a critical carbon source for *Lactobacillus* species, which metabolize glycogen into lactic acid (Amabebe and Anumba, 2018; Hertzberger et al., 2022). The resulting acidic environment (pH <4.5) inhibits potentially pathogenic microorganisms, serving as a natural defense against infection.

Compared with non-pregnant women, pregnant women typically exhibit reduced microbial diversity and a shift in community composition (Aagaard et al., 2012). Vaginal communities are often dominated by *Lactobacillus* spp., with minor contributions from *Actinomycetales*, *Clostridiales*, and *Bacteroidales*, whereas non-pregnant women harbor a broader microbial spectrum, including *Prevotella*, *Streptococcus*, *Bifidobacteriaceae*, *Burkholderiales*, and *Veillonellaceae*. *Lactobacillus* abundance is generally higher in pregnancy, while Mollicutes and Ureaplasma prevalence is lower; overall, the microbial community is more stable but less diverse (Romero et al., 2014). Despite this general stability, physiological changes can predispose some women to dysbiosis. Elevated estrogen levels promote vaginal secretions and glycogen accumulation, potentially favoring overgrowth of specific microbes (Nugent et al., 1991). Increased vascularity, mucosal edema, and higher epithelial permeability may compromise the mucosal barrier. Immunologically, systemic and local modulation during pregnancy can reduce the vagina’s capacity to resist microbial imbalance, increasing susceptibility to infections, which in turn may contribute to adverse outcomes such as preterm labor and premature rupture of membranes. Understanding these microbial and physiological dynamics is essential for anticipating complications and guiding preventive strategies.

Research has demonstrated significant variations in the vaginal microbiota during full-term pregnancy and delivery. Dominguez-Bello et al. (2010) used 454-pyrosequencing to analyze the vaginal microbiota of nine full-term Indigenous women before labor onset and during active labor, revealing considerable individual differences, particularly among *Lactobacillus* species. The study also highlighted a strong correlation between neonatal gut colonization and delivery mode, underscoring the dynamic nature of maternal microbiota and its influence on early neonatal microbial development.

Longitudinal studies indicate that the vaginal microbiota undergoes dynamic changes before, during, and after pregnancy. Analyses using 16S rRNA sequencing show that *Lactobacillus* remains the dominant genus prior to and during gestation, with the overall community largely stable (Hu et al., 2023). A large-scale longitudinal study of healthy Chinese women reported significant shifts in vaginal pH, microbial diversity, dominant taxa, and Nugent scores from preconception to pregnancy; nonetheless, most women maintained a normal or mildly imbalanced, *Lactobacilli*-dominated microbiota (Li et al., 2025). These findings suggest that pregnancy is associated with a stabilized, lactobacilli-predominant vaginal ecosystem, supporting mucosal barrier integrity, inhibiting pathogen colonization, and reducing the risk of ascending infections.

While pregnancy is associated with microbial stability, the transition to the postpartum period is marked by pronounced changes in community composition. *Lactobacillus crispatus* predominates during pregnancy, but postpartum communities exhibit increased diversity, a relative decline in lactobacilli, and expansion of taxa such as *Streptococcus anginosus* and *Prevotella bivia* (Nunn et al., 2021). Biochemical properties of vaginal secretions also change, including elevated hyaluronan and Hsp70 levels and decreased D- and L-lactate, likely reflecting postpartum alterations in pH, immune status, and nutrient availability. These shifts make the previously stable, lactobacilli-dominated ecosystem more vulnerable to disruption.

### 2.2 The gut microbiome

As a crucial immunoregulatory organ, the gut microbiota dynamically interacts with the host through metabolic products and immune signaling molecules. These interactions not only help maintain intestinal barrier integrity but also play a key role in regulating systemic immune homeostasis. Studies have shown that maternal gut microbiota is closely associated with both maternal and fetal/neonatal health (Dicks et al., 2018; Wang et al., 2018; Ma et al., 2023). Therefore, investigating the gut microbiota during pregnancy is of particular importance.

During pregnancy, the maternal physiological state undergoes significant changes due to hormonal, immunological, and metabolic alterations. To support maternal health and fetal development throughout gestation, the diversity and relative abundance of the maternal gut microbiota undergo corresponding adjustments (Collado et al., 2008). These changes exhibit distinct stage-specific patterns. In early pregnancy, the gut microbiota of pregnant women resembles that of non-pregnant women. However, as gestation progresses, notable shifts occur, including an increase in the abundance of bacteria belonging to the phyla *Actinobacteria* and *Proteobacteria*, accompanied by a reduction in alpha diversity and an increase in beta diversity. Notably, the rise in *Proteobacteria*, which are major contributors to inflammation, has been closely associated with intestinal inflammation. Concurrently, a marked decrease in *Faecalibacterium*-a genus of butyrate-producing bacteria with anti-inflammatory properties-has been observed (Koren et al., 2012; Mukhopadhya et al., 2012). These microbial shifts suggest a pro-inflammatory state of the gut microbiota in late pregnancy. Further studies have found that inflammatory markers in maternal feces increase throughout gestation, with mild inflammation observed in the intestinal epithelium, especially during the third trimester.

Longitudinal studies have demonstrated that both maternal gut microbiota and metabolome undergo dynamic changes throughout pregnancy (Pan et al., 2024). The microbial composition is notably reshaped, with the *Lachnospiraceae* and *Ruminococcaceae* families showing the most pronounced shifts, particularly the *Lachnospiraceae FCS020* group and *Ruminococcaceae UCG-003*. Concurrently, key metabolites in maternal serum and feces, including nutrients essential for fetal development, anti-inflammatory compounds, and steroid hormones, also fluctuate, suggesting a tight link between microbiota dynamics and host metabolic reprogramming. Another study reported an increase in the Bacteroidetes/Firmicutes ratio from early to late pregnancy (Gao et al., 2024). At the genus level, several taxa show increased abundance in mid-to-late gestation, such as *Bilophila*, while the rise of *Mitsuokella*, *Clostridium sensu stricto*, and *Weissella* may be associated with elevated maternal fasting glucose. Additionally, the abundances of *Corynebacterium*, *Rothia*, and *Granulicatella* were linked to dyslipidemia during pregnancy. These findings indicate that, during normal gestation, not only the overall microbial structure (e.g., phylum-level ratios) changes, but specific genera also dynamically fluctuate in association with maternal metabolic status, highlighting the microbiota–metabolome–host interactions. Multinational longitudinal cohort studies further confirmed that from early to late pregnancy, maternal gut microbiota α-diversity (richness) declines significantly, and β-diversity (community composition) changes markedly (Tang et al., 2022). At the genus level, some unclassified *Lachnospiraceae* and *Ruminococcaceae* show decreasing trends across different regions, suggesting that gut microbial remodeling during pregnancy may follow general patterns while also being influenced by geography, lifestyle, and individual variation.

Interestingly, the gut microbiota composition in late pregnancy resembles that found in individuals with obesity, particularly in terms of enhanced microbial energy-harvesting capacity (Greenblum et al., 2012). This is closely linked to the metabolic characteristics of late pregnancy, which is considered a diabetogenic state. This state is marked by sustained maternal hyperglycemia to ensure continuous nutrient supply to the fetus (Tilg and Moschen, 2006). Additionally, increased maternal fat accumulation serves as an energy reserve for lactation. This metabolic adaptation is accompanied by elevated levels of pro-inflammatory cytokines and is strongly associated with insulin resistance, suggesting the involvement of shared regulatory mechanisms. From late pregnancy to the postpartum period, changes in the gut microbiota become relatively minor, and its overall composition remains relatively stable (Jost et al., 2014).

### 2.3 The oral microbiomes

The oral microbiota represents the second largest microbial ecosystem in the human body, comprising over 700 distinct bacterial species (Barbour et al., 2022). Its composition is influenced by various factors, including nutrition, oxygen levels, and pH (Saadaoui et al., 2021). From birth, these microorganisms establish a close symbiotic relationship with the host and, through long-term evolution and adaptation, develop into a diverse and dynamic ecological system. Studies have demonstrated that the oral microbiome undergoes significant changes across different stages of life, particularly during the transitions from infancy to adolescence and adulthood (Dzidic et al., 2018; Corrêa et al., 2025; Olate et al., 2025). During pregnancy, women experience substantial hormonal, metabolic, and immunological changes, which profoundly affect the oral microenvironment and microbial composition. Notably, elevated progesterone levels increase susceptibility to dental plaque, contributing to the development of pregnancy gingivitis (Yang et al., 2024; Zhao et al., 2024). Porphyromonas gingivalis, a key periodontal pathogen, has been found to be positively correlated with progesterone levels in early pregnancy and may, in turn, promote further elevation of progesterone (Kornman and Loesche, 1980; Massoni et al., 2019; Kato et al., 2022). In addition, hormonal fluctuations during pregnancy facilitate the proliferation of certain anaerobic Gram-negative bacteria, particularly *Prevotella intermedia*, *Campylobacter rectus*, and *Prevotella nigrescens*, which are commonly associated with periodontal diseases and oral inflammation (Jensen et al., 1981; Muramatsu and Takaesu, 1994; Yokoyama et al., 2008; Gürsoy et al., 2009). High levels of estrogen have also been shown to increase the risk of Candida infections (Kumar et al., 2013; Fujiwara et al., 2017). These changes collectively contribute to the overgrowth of specific pathogenic microbes, disrupting the oral microbial balance and exacerbating the complexity of oral health issues during pregnancy.

Compared to non-pregnant women, the total number of oral microorganisms significantly increases during all stages of pregnancy, particularly in early pregnancy (Fujiwara et al., 2017). Lin et al. reported that the salivary microbiota of pregnant women exhibited a significantly higher Shannon diversity index than that of non-pregnant women (Lin et al., 2018). In addition, the levels of *Porphyromonas gingivalis* and *Aggregatibacter actinomycetemcomitans* were markedly elevated in the gingival crevices of women during early and mid-pregnancy. In late pregnancy, there was also a notable increase in *Candida* species (Fujiwara et al., 2017). Studies analyzing bacterial community composition in salivary and subgingival plaque (SGP) samples from pregnant women have identified *Firmicutes*, *Bacteroidetes*, and *Actinobacteria* as the predominant phyla during pregnancy (Balan et al., 2018). In SGP samples, *Prevotella*, *Fusobacterium*, *Streptococcus*, *Veillonella*, and *Terrahaemophilus* were found in high abundance. In saliva, *Prevotella*, *Streptococcus*, *Veillonella*, *Neisseria*, and *Terrahaemophilus* were the dominant genera. These findings suggest that the composition of the oral microbiota undergoes significant changes during pregnancy, with specific bacterial taxa increasing in both abundance and activity as gestation progresses. It is worth noting that alterations in the oral microbiota during pregnancy are part of the physiological changes associated with gestation. However, these changes in the oral microenvironment and its microbial communities make pregnant women more susceptible to periodontal diseases (Balan et al., 2018). Studies have shown that dysbiosis of the oral microbiota, when combined with gingival inflammation, may contribute to adverse pregnancy outcomes, including miscarriage, PTB, low birth weight, and gestational hypertension (Boggess et al., 2003; Moore et al., 2004).

## 3 The microbiome’s role in preterm birth

### 3.1 Vaginal microbiome and preterm birth

**Figure 1:**
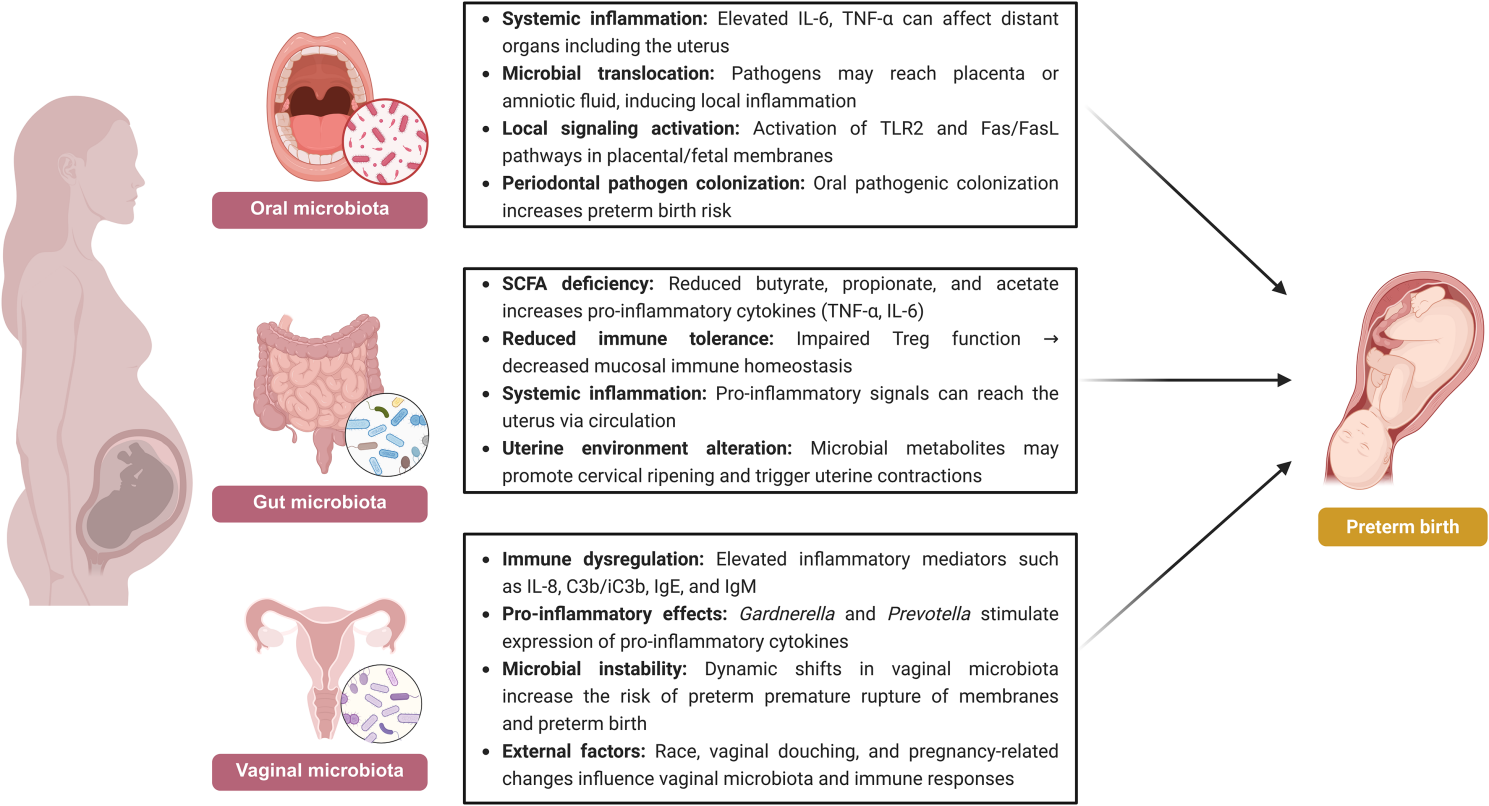
Schematic representation of the role of vaginal, gut, and oral microbiomes in preterm birth. This figure was created with BioRender.com (https://www.biorender.com).

A vaginal microbiota dominated by *Lactobacillus* with low diversity is considered a “healthy” state. In contrast, when the abundance of *Lactobacillus* decreases and anaerobic bacteria such as *Gardnerella* and *Prevotella* increase, microbial diversity rises, which leads to dysbiosis. Studies have shown that women who experience preterm birth typically exhibit higher vaginal microbial diversity, a characteristic often associated with a “low-*Lactobacillus*” microbiota profile (Gupta et al., 2019). Additionally, research has found that significant declines in richness, diversity, and evenness of the vaginal microbiota from early to mid-pregnancy are linked to a higher risk of preterm birth (Stout et al., 2017). Compared to women who deliver at term, those who deliver preterm tend to have a more unstable vaginal microbiome. Although no specific bacterial strain has been directly linked to preterm birth, dynamic changes in the vaginal microbiota during early pregnancy may play a key role in pregnancy outcomes, offering a potential window for early warning and intervention. Recent studies have investigated the relationship between microbiota dysbiosis and preterm birth (**Table 1**). The proposed mechanisms of microbiota-associated preterm birth are summarized in **Figure 1**.

**Table 1:** Recent studies on microbiota dysbiosis and preterm birth.

Author (Year)	Sample size	Sample site	Changes in microbiota	References
Celik et al., 2025	Short cervix: 31; Control: 27	Vaginal Microbiome	Short cervix: higher species diversity (Shannon & Chao indices), decreased *Lactobacillus*, reduced *Firmicutes*, increased *Bacteroidota* and *Proteobacteria*. *Lactobacillus gasseri* lower; *Lactobacillus crispatus* declined. Vaginal progesterone did not significantly alter microbiota.	(Celik et al., 2025)
Lee et al., 2025	Short cervix: 35; Control: 12	Vaginal Microbiome	Decreased *Lactobacillus* dominance, higher diversity; enrichment of community state type IV. Spontaneous preterm birth: more opportunistic pathogens; term birth: more *Lactobacillus*-associated taxa.	(Lee et al., 2025)
Wang et al., 2025	195	Vaginal Microbiome	Advanced maternal age with cervical columnar ectopy: increased *Gardnerella vaginalis*, reduced *Lactobacillus crispatus*, disrupted galactose metabolism. *Lactobacillus crispatus* galactosidase protective; *Gardnerella vaginalis* associated with adverse outcomes including preterm birth.	(Wang et al., 2025)
Nam et al., 2025	Term: 40; Preterm: 20	Vaginal Microbiome	Term: *Lactobacillus crispatus* dominant; Preterm: higher diversity, increased *Prevotella salivae* and *Ureaplasma*. Shifts correlated with shorter cervical length.	(Nam et al., 2025)
Jiang et al., 2025	Term: 99; Preterm: 53	Vaginal Microbiome	Recurrent spontaneous preterm birth: early *Lactobacillus iners* dominance → shift to non-Lactobacillus community state type IVA/IVB. Non-recurrent: *Lactobacillus crispatus* dominant. *Lactobacillus iners* dominance linked to higher recurrence risk.	(Jiang et al., 2025)
Shen et al., 2025	Preterm: 23; Full-term: 54	Vaginal Microbiome	Preterm: lower species diversity (Shannon 2.65 vs. 3.56), increased *Firmicutes*, predominance of *Lactobacillus jensenii* (negatively correlated with gestational age). Full-term: higher diversity, balanced *Lactobacillus* composition. Microbiota-metabolite-inflammation links suggest dysbiosis drives inflammation-induced preterm birth.	(Shen et al., 2025)
Zhang et al., 2025	Preterm: 36; Term: 29	Vaginal Microbiome	Preterm: lower diversity (ACE, Chao1, Simpson, Shannon), decreased *Lactobacillus*, increased *Gardnerella*, *Atopobium*, *Ralstonia*, *Sneathia*. Term: higher diversity, *Lactobacillus*-dominant. Certain bacteria potential PTB predictors.	(Zhang et al., 2025)
Vinodhini et al., 2025	Preterm: 98; Term: 98	Vaginal Microbiome	Spontaneous preterm labor: higher frequency of vaginal infections; Gram-negative bacilli most common, mostly sensitive to third-generation cephalosporins.	(Vinodhini et al., 2025)
Miao et al., 2025	Discovery: 4,286; Validation: 1,027	Gut Microbiome	Early-pregnancy gut microbiota linked to preterm birth. *Clostridium innocuum* strongest predictor. Microbial risk score combining taxa predicts PTB risk, interacts with maternal polygenic risk. *Clostridium innocuum* degrades 17β-estradiol and progesterone, affecting hormones. Higher levels associated with poor sleep, pre-pregnancy vaginitis, early hormone use.	(Miao et al., 2025)
Song et al., 2025	Oral: 109; Vaginal: 93; Gut: 37	Oral, vaginal, and gut microbiota	Threatened preterm birth: oral microbiota imbalance; vaginal microbiota altered with decreased *Lactobacillus*; gut microbiota overall structure similar to controls.	(Song et al., 2025)
Park et al., 2024	Preterm: 30; Full-term: 30	Oral Microbiome	Preterm: lower diversity, weaker microbial network, higher *Actinomycetales*. Full-term: higher *Spirochaetes*, *Treponema*, *Porphyromonas*, stronger connectivity.	(Park et al., 2024)
Liao et al., 2023	Preterm: 40; Term: 135	Vaginal Microbiome	Preterm: higher genetic diversity, richer antimicrobial resistance genes; *Gardnerella* drove diversity, frequent recombination, stronger selection in lipid metabolism genes. Term: lower diversity.	(Liao et al., 2023)
Vidmar Šimic et al., 2023	Preterm: 61; Term: 91	Oral Microbiome	Preterm: increased Firmicutes, Bacteroidetes; decreased Proteobacteria; enriched *Veillonella*, *Prevotella*, *Capnocytophaga*, notably *Veillonella massillensis*. Term: lower abundance of these genera.	(Vidmar Šimic et al., 2023)
Yang et al., 2023	43 mothers, 80 gut samples	Gut Microbiome	Preterm: lower diversity, gut microbiota reorganized; reduction of short-chain fatty acid-producing bacteria, especially *Lachnospiraceae*, *Ruminococcaceae*, *Eubacteriaceae*. *Lachnospiraceae* contributed most to species and metabolic pathway differences.	(Yang et al., 2023)
Yin et al., 2021	Threatened preterm labor: 19; Controls: 22	Gut Microbiome	Preterm: gut dysbiosis; enrichment of oral-origin bacteria (*Porphyromonas*, *Streptococcus*, *Fusobacterium*, *Veillonella*); depletion of gut commensals (*Coprococcus*, *Gemmiger*).	(Yin et al., 2021)

Multiple studies have demonstrated the protective role of *Lactobacillus*, whereas a “low-*Lactobacillus*” vaginal microbiota may increase the risk of spontaneous PTB (Bayar et al., 2020; Bennett et al., 2020; Tsonis et al., 2020). Key anaerobic genera such as *Gardnerella* and *Prevotella* are particularly enriched in these dysbiotic profiles. They can induce pro-inflammatory cytokine expression and are frequently present in patients with PPROM (Tsonis et al., 2020). Other studies have also observed significantly increased abundances of these two pathogenic genera in the vaginal microbiota of women who experienced preterm birth (Li et al., 2024). Further research has used immune factor clustering to classify pregnant women into three immunological subtypes, revealing that the high inflammatory response subtype is significantly associated with an increased risk of preterm birth (Liang et al., 2024). Significant differences in the diversity and composition of the vaginal microbiota have been found among these immune subtypes, with downregulation of certain microbial functional pathways in the high-risk group. Factors such as C3b/iC3b, IL-8, IgE, and IgM may mediate preterm birth. *Lactobacillus crispatus* may exert a protective effect by modulating maternal immune responses. These findings suggest that the interaction between the vaginal microbiota and the immune system during mid-pregnancy plays a crucial role in the pathogenesis of preterm birth.

A large-scale nested case-control study involving 824 women from diverse racial backgrounds further validated the association between the vaginal microbiome and spontaneous PTB (Sun et al., 2022). The study found that African American women exhibited higher vaginal α-diversity, higher abundance of *Lactobacillus* iners, and lower abundance of *Lactobacillus crispatus*. Among women who did not practice vaginal douching, a lower abundance of *Lactobacillus crispatus* was significantly associated with an increased risk of spontaneous PTB; specifically, each tenfold increase in its relative abundance was linked to an approximately 20% reduction in spontaneous PTB risk. Moreover, vaginal douching attenuated the associations between microbiota composition, race, and spontaneous PTB, likely because it disrupts the protective vaginal environment by reducing *Lactobacilli*, altering pH, and promoting colonization by anaerobic bacteria. Differences in vaginal microbiota across racial groups may reflect not only genetic and immunological variations but also social determinants such as diet, stress, hygiene practices, and healthcare access. These factors can influence the vaginal microenvironment and immune responses, thereby affecting susceptibility to dysbiosis and preterm birth. Therefore, while the vaginal microbiome holds promise as a biomarker for preterm birth risk, understanding the biological and social mechanisms underlying these differential effects is crucial for interpreting population-specific risks.

Studies have shown that, compared with full-term pregnancies, women with PPROM exhibit a greater prevalence of vaginal dysbiosis even before membrane rupture, characterized primarily by a reduction in *Lactobacillus* species and an increase in potentially pathogenic bacteria such as *Sneathia* (Brown et al., 2018). Notably, this microbial imbalance can persist after membrane rupture. Furthermore, erythromycin-commonly used as a prophylactic antibiotic in PPROM-may exacerbate dysbiosis, particularly in individuals whose vaginal microbiota is initially dominated by *Lactobacillus*. This antibiotic-induced shift is closely associated with an increased risk of funisitis and early-onset neonatal sepsis. These findings suggest that the vaginal microbiota not only plays a role in the pathogenesis of PPROM but may also influence maternal and neonatal outcomes. Importantly, the study highlights that traditional prophylactic antibiotic regimens, while widely used, may in some cases worsen microbial imbalances, potentially introducing new risks. Therefore, this research underscores both the predictive value and modifiable nature of the vaginal microbiome as a prenatal risk factor and calls for a reevaluation of current antibiotic prophylaxis strategies in the context of PPROM.

### 3.2 Gut microbiome and preterm birth

Although traditional research has primarily focused on the association between the vaginal microbiota and PTB, increasing attention has recently been given to the potential role of the gut microbiota in pregnancy outcomes. Studies have shown that pregnant women with inflammatory bowel disease have a significantly higher risk of premature rupture of membranes and PTB (Boyd et al., 2015), suggesting that gut microbial dysbiosis may contribute to adverse pregnancy outcomes by affecting systemic inflammation or immune regulation. On the other hand, pregnant women who maintain healthy dietary habits-such as consuming adequate amounts of vegetables, fruits, and whole grains-have a significantly lower incidence of PTB. This may be closely related to the positive influence of diet on the composition and function of the gut microbiota (Chia et al., 2019). Therefore, disruption of gut microbial homeostasis during pregnancy may be an important risk factor for PTB.

Studies have shown that pregnant women who experience PTB exhibit significantly lower gut microbial α-diversity compared to those who deliver at term, with a marked reduction in the abundance of key beneficial bacteria such as *Bifidobacterium*, *Streptococcus*, *Clostridium cluster*, and *Bacteroides* (Gershuni et al., 2021). Among them, *Bifidobacterium* and *Streptococcus* are major producers of short-chain fatty acids (SCFAs), including butyrate, propionate, and acetate. SCFAs play a protective role in pregnancy by alleviating systemic inflammation through the suppression of pro-inflammatory cytokines such as TNF-α and IL-6, and by inhibiting enzymes involved in uterine remodeling and fetal membrane degradation, such as matrix metalloproteinases (MMPs). The depletion of these key microbial populations therefore not only reduces SCFA-mediated anti-inflammatory and barrier-protective effects, but may also impair immune regulation, potentially triggering premature uterine activation and contributing to PTB (Fukuda et al., 2011; Moylan et al., 2020). In addition, bacteria such as *Bifidobacterium*, *Clostridium*, and *Bacteroides* can promote the secretion of anti-inflammatory cytokines-such as TGF-β and IL-10-which activate Tregs, enhance immune tolerance of the intestinal mucosa, and help maintain immune homeostasis during pregnancy [68,69].

A study utilizing 16S rRNA amplification combined with terminal restriction fragment length polymorphism (T-RFLP) technology found that in pregnant women who experienced PTB, the abundance of *Clostridium clusters* XVIII, IV, and XIVa, as well as *Bacteroides*, was significantly reduced, whereas the abundance of *Lactobacillales* was elevated (Shiozaki et al., 2014). In addition, a study based on the Norwegian NoMIC cohort further explored the association between maternal gut microbiota and PTB. Among 121 women who delivered vaginally at term, fecal samples were collected on the fourth day postpartum, and microbial composition and diversity were analyzed using 16S rRNA sequencing (Dahl et al., 2017). The results revealed that lower gut microbial Shannon diversity was significantly associated with spontaneous PTB: for each interquartile range increase in diversity, the risk of PTB decreased by approximately 38%. After adjusting for multiple confounding factors, this risk reduction increased to 48%. Moreover, abundances of *Bifidobacterium*, *Streptococcus*, and *Clostridia* were markedly reduced in the PTB group. These findings suggest that both overall maternal gut microbial diversity and the abundance of specific beneficial taxa play regulatory roles in the occurrence of PTB.

However, the mechanisms by which the gut microbiota influences the uterine environment and contributes to preterm labor remain incompletely understood. Traditionally, the intrauterine environment was considered sterile, but increasing clinical evidence indicates that microbial colonization may exist even under physiological conditions (Aagaard et al., 2014; Zheng et al., 2015; Bassols et al., 2016; Xie et al., 2025). Intrauterine microbes may reach the uterus through multiple routes, including ascending transmission from the vagina and cervix, as well as translocation from non-reproductive sites such as the oral cavity or gastrointestinal tract. Metabolites produced by these distant microbial communities can enter the bloodstream and act on the cervix and uterine tissues, promoting cervical ripening and the initiation of uterine contractions, thereby potentially contributing to the onset of PTB.

In summary, gut microbial dysbiosis may contribute to PTB through multiple interrelated mechanisms, including reduction of beneficial bacteria and SCFA production, immune dysregulation, increased pro-inflammatory signaling, and systemic effects of microbial metabolites on the uterus. These mechanistic insights suggest that maintaining gut microbial homeostasis may be a critical factor in preventing PTB.

### 3.3 Oral microbiome and preterm birth

Although most research on microbiota and PTB has focused on the vaginal and gut microbiomes, growing evidence suggests that the oral microbiome may also play a significant role in pregnancy outcomes. Hormonal, metabolic, and immunological changes during pregnancy can profoundly affect the oral microenvironment, leading to alterations in microbial composition and increased susceptibility to periodontal disease. During pregnancy, rising levels of estrogen and progesterone significantly increase the abundance of periodontal pathogens and opportunistic bacteria in the oral cavity, resulting in a typical “pathogenic shift.” This microbial shift notably elevates the risk of gingivitis and periodontitis in pregnant women (Lin et al., 2018). Among these, *Porphyromonas gingivalis*, *Prevotella*, and *Mogibacterium* species become particularly abundant during the second and third trimesters and tend to return to pre-pregnancy levels after childbirth (Balan et al., 2018, 2021). Additionally, pregnancy significantly alters the alpha diversity of the oral microbiome. Studies have shown that the Shannon diversity index of dental plaque is higher in women during late pregnancy than in non-pregnant women, indicating increased richness and diversity of the oral microbial community (Lin et al., 2018; Balan et al., 2021).

Several clinical studies have detected periodontal pathogens such as *Porphyromonas gingivalis* and *Fusobacterium nucleatum* in the placenta and amniotic fluid of women who experienced PTB, suggesting that oral bacteria may translocate to the placenta via the bloodstream, directly triggering local inflammatory responses and contributing to preterm delivery (León et al., 2007; Ercan et al., 2013). Animal studies further support this mechanism: following oral infection with *Porphyromonas gingivalis* and *Fusobacterium nucleatum*, these pathogens have been detected in placental tissues, accompanied by elevated serum levels of inflammatory cytokines such as tumor necrosis TNF-α, IL-17, and IL-6. Increased expression of Fas/FasL signaling pathways and Toll-like receptor 2 (TLR2) in placental tissue has also been observed, ultimately contributing to a higher risk of PTB and low birth weight (Han et al., 2004; Ao et al., 2015; Liang et al., 2018). Mechanistically, these pathogens can adhere to and invade placental or fetal membranes via fimbriae and other virulence factors. Additionally, microbial products such as lipopolysaccharides (LPS) can activate maternal immune cells, inducing systemic inflammation and cytokine release, which may promote cervical ripening, membrane weakening, and initiation of uterine contractions (Hasegawa-Nakamura et al., 2011; Cappelletti et al., 2020; Terzic et al., 2021).

Moreover, a reduction in the oral commensal *Lautropia mirabilis*, along with an enrichment of *Prevotella melaninogenica*, may be closely associated with spontaneous PTB, highlighting the combined effect of dysbiosis and pathogenic invasion (Yang et al., 2022). Dysbiosis of the oral microbiome during pregnancy is associated with elevated levels of inflammatory mediators, including IL-6 and TNF-α, which can enter systemic circulation and affect distant organs such as the uterus. Certain pathogens may also translocate via the bloodstream and colonize the placenta or amniotic fluid, inducing local inflammation that promotes the onset of labor.

Taken together, current evidence suggests that oral microbial imbalance, particularly in the context of periodontal disease, may increase the risk of PTB through systemic inflammation, microbial translocation, and disruption of the commensal microbial balance. Therefore, maintaining good oral hygiene and proactively managing periodontal disease may represent important yet often overlooked strategies for preventing PTB.

## 4 Therapeutic strategies: targeting the microbiome in PTB

Probiotics, as key regulators of the microbiome, have been widely applied in clinical practice, particularly for maintaining gut health, but their role in preventing PTB remains controversial. Different *Lactobacillus* species may have distinct effects on pregnancy outcomes, and the optimal strains for PTB prevention are not fully established. Some evidence suggests that targeted probiotic supplementation may reduce the risk of PTB related to infection and inflammation. For example, women who consumed *Lactobacillus rhamnosus* NCC 4007 (CGMCC 1.3724) and *Bifidobacterium animalis* subsp. *lactis* NCC 2818 (CNCM I-3446) during the preconception period showed a significantly reduced risk of PTB (Godfrey et al., 2021). Myhre et al. (2013) conducted a large cohort study including 18,888 women and found that consuming probiotic-rich foods during early to mid-pregnancy reduced the incidence of PTB by approximately 18%. Additionally, in an LPS-induced PTB mouse model, pretreatment with *Lactobacillus rhamnosus* GR-1 culture supernatant reduced the rate of PTB by 43% (Yang et al., 2014). Simultaneously, levels of inflammatory and chemotactic factors in the serum, placenta, myometrium, and amniotic fluid of these mice were significantly decreased, further supporting the notion that modulation of the gut microbiota can influence maternal inflammation and improve pregnancy outcomes.

Moreover, probiotics may restore a healthy vaginal microbiota, reduce colonization by pathogens, and regulate immune responses, thereby lowering the risk of premature rupture of membranes and cervical insufficiency (Vaduva et al., 2024). BV is an important risk factor for PTB, and conventional antibiotic treatments have limited efficacy in reducing PTB rates. BV is typically characterized by a depletion of vaginal *Lactobacillus*, leading to microbial imbalance. Consequently, probiotic therapy aims to reestablish *Lactobacillus* dominance, thereby restoring a healthy vaginal microecological environment and ultimately increasing the likelihood of full-term pregnancy (Reid and Bocking, 2003). Probiotics can successfully colonize the vagina, inhibit the growth of pathogens such as *Gardnerella* and *Escherichia coli*, and modulate local immune responses to mitigate inflammatory cascades, thereby effectively interrupting inflammation-driven mechanisms that trigger PTB.

It is noteworthy that erythromycin, commonly used as a prophylactic antibiotic to prevent complications of PPROM, although effective in reducing infection risk, may exacerbate vaginal dysbiosis, especially in individuals whose vaginal microbiota is originally dominated by *Lactobacillus* (Brown et al., 2018). To address concerns regarding antibiotic-induced dysbiosis, studies have evaluated the adjunctive use of vaginal probiotics alongside antibiotics for the prevention and treatment of PPROM (Baradwan et al., 2023). The results showed that the combination therapy group had significantly longer gestational age at delivery and latency period compared to the antibiotic-only group; furthermore, the rates of neonatal admission to the neonatal intensive care unit (NICU) and hospital stay durations were significantly reduced, and neonatal birth weights were significantly increased.

Despite these promising findings, several challenges remain, including strain-specific efficacy, optimal dosage and timing, inter-individual variability in maternal microbiota, and the need for long-term safety data. Future research should focus on identifying the most effective *Lactobacillus* strains, elucidating how probiotics modulate maternal immune and inflammatory pathways, and conducting large-scale, well-designed clinical trials to establish standardized supplementation protocols and integrate multi-omics approaches to predict individual responses and optimize therapeutic outcomes.

## 5 Conclusions and perspectives

During pregnancy, the maternal microbiome undergoes significant, niche-specific changes driven by hormonal, metabolic, and immune shifts. The vaginal microbiome becomes more stable and dominated by *Lactobacillus* species, creating an acidic environment that protects against infections. However, in some women, hormonal and immune alterations may predispose them to dysbiosis, increasing the risk of bacterial vaginosis and adverse outcomes such as PTB. The gut microbiota also displays stage-specific shifts, with late pregnancy marked by reduced diversity, increased *Proteobacteria*, and a pro-inflammatory profile resembling that of metabolic syndrome. These changes reflect the host’s metabolic adaptation to support fetal development but may also contribute to systemic inflammation and insulin resistance. The oral microbiota undergoes profound changes as well, with elevated progesterone and estrogen levels promoting gingival inflammation and overgrowth of periodontal pathogens. Oral dysbiosis during pregnancy has been linked to adverse outcomes, including miscarriage, PTB, and low birth weight.

The maternal microbiome plays a key role in the pathogenesis of PTB. Vaginal dysbiosis, marked by reduced *Lactobacillus* and increased anaerobes like *Gardnerella* and *Prevotella*, is strongly linked to spontaneous PTB and PPROM, with immune and socio-environmental factors further shaping risk. The gut microbiota contributes through its impact on inflammation and immune regulation. Loss of SCFA-producing bacteria such as *Bifidobacterium* and *Clostridium* may impair immune balance and increase PTB risk. Reduced gut microbial diversity has also emerged as a potential biomarker. Although less studied, the oral microbiome also shifts during pregnancy. Increased periodontal pathogens and their translocation to the placenta may trigger inflammatory responses, contributing to adverse outcomes such as PTB.

Probiotics show promise in reducing PTB risk by modulating maternal gut and vaginal microbiota, thereby lowering infection and inflammation. Evidence from cohort studies and animal models supports their role in improving pregnancy outcomes through restoring microbial balance and regulating immune responses. In the vaginal environment, probiotics help reestablish *Lactobacillus* dominance, inhibit pathogens, and reduce risks such as bacterial vaginosis and premature membrane rupture. While antibiotics such as erythromycin are standard for PPROM, they may worsen vaginal dysbiosis. Combining probiotics with antibiotics appears to improve perinatal outcomes, though further high-quality trials are needed.

Future research should clarify optimal probiotic strains, timing, and mechanisms, with an emphasis on integrating multi-omics data, including metagenomics and metabolomics, to comprehensively characterize functional alterations in the maternal microbiome and their impact on preterm birth. This approach will enhance mechanistic understanding and inform targeted, evidence-based interventions to reduce PTB risk.
